# The effectiveness of a Mindful Yoga and Common Yoga intervention on emotion regulation and well-being of adolescents; A nonrandomized quasi-experimental study

**DOI:** 10.1371/journal.pone.0355375

**Published:** 2026-08-26

**Authors:** Jobin K, Lena Ashok, M. Manjula, Teddy Andrews J. J, Vani Lakshmi R, Soyuz John

**Affiliations:** 1 Department of Social and Health Innovation, Prasanna School of Public Health, Manipal Academy of Higher Education, Manipal, India; 2 Department of Clinical Psychology, NIMHANS, Bangalore, India; 3 Department of Health Technology and Informatics, Prasanna School of Public Health, Manipal Academy of Higher Education, Manipal, India; 4 Department of Psychiatry, Kasturba Medical College, Manipal Academy of Higher Education, Manipal, India; Japanese Academy of Health and Practice, JAPAN

## Abstract

**Objectives:**

Yoga programs have gained wide attention in school settings to promote adolescent mental health. However, comparative evaluations of distinct yoga-based approaches are limited. This study examined the effectiveness of a Mindful Yoga and Meditation (MYM) program and the Common Yoga Protocol (CYP) in reducing psychological distress and enhancing mindfulness, emotion regulation, and overall well-being among secondary school students in India.

**Methods:**

The study employed a quasi-experimental design. A total of 147 students were assigned to three groups: MYM, CYP, and waitlist-control. Both interventions were conducted over 21 days, with assessments at baseline (T1), post-intervention (T2), and 3-month follow-up (T3). The outcome measures included psychological distress (K10), mindfulness (CAMM), emotion regulation (ERQ-CA), and well-being (WHO-5). A linear mixed model was employed to examine the main effects of group and time, as well as the group × time interaction, across all outcome measures.

**Results:**

Both the MYM and CYP groups showed significant improvements compared to the control group. The MYM participants demonstrated greater and more sustained gains in mindfulness (*p* < .001), cognitive reappraisal (*p* = .026), and well-being (*p* < .001), along with larger reductions in distress (*p* < .001) and expressive suppression (*p* < .001). While CYP was effective, its benefits were comparatively smaller and less durable when compared to MYM. No significant changes were observed in the control group.

**Conclusion:**

The findings support the feasibility and effectiveness of brief school-based yoga interventions in promoting adolescent mental health. Importantly, integrating mindfulness into yoga practices (as in MYM) yielded superior outcomes, emphasizing the added value of mindful components.

## Introduction

Adolescence is a critical developmental period marked by rapid biological, emotional, and social changes, often accompanied by increased vulnerability to psychological distress [1]. Difficulties in emotion regulation during this stage can significantly impact mental health, interpersonal functioning, and academic outcomes and are known to contribute to the onset of psychiatric disorders later in life [2]. Difficulties in emotion regulation can increase the risk of developing psychological problems in adulthood, as many mental health issues have their origins in childhood or adolescence. Given that many mental health conditions originate during adolescence, strengthening emotion regulation is a key target for early intervention and long-term psychological well-being [3].

India has the largest adolescent population globally [4]. According to the WHO, one in seven adolescents globally experiences a mental health disorder [5]. The COVID-19 pandemic has further exacerbated these challenges, adding additional layers of stress and uncertainty [6]. The increasing prevalence of emotion dysregulation among adolescents underscores the urgent need for early interventions to prevent the risk of developing psychiatric disorders later in adolescence and adulthood [7]. Mindfulness and Yoga are well-established interventions for promoting mental health and overall well-being [8–11].

Mindfulness and yoga have emerged as promising evidence-based approaches to enhance emotional regulation and overall mental health. “Mindfulness is widely defined as the ability to bring one’s complete attention to the experiences occurring in the present moment, in a non-judgmental or accepting way” [12]. The most commonly practiced style of yoga is Hatha Yoga, which integrates physical postures (āsana), breathing practices (prāṇāyāma), and meditative awareness (dhyāna), providing a holistic approach to fostering self-regulation in adolescents [13,14]. Mindful yoga, which combines mindfulness principles with yoga practice, has gained increasing attention for its capacity to simultaneously engage cognitive, emotional and bodily processes. Emerging evidence suggests that mindful yoga may produce greater improvements in psychological well-being than other mindfulness practices, highlighting its potential as an integrated intervention for adolescents [15–18].

Previous research has proven that mindfulness is highly correlated with positive indicators of mental health and well-being [19–23]. There are various mindfulness-based training programs that incorporate different mindfulness techniques, making it challenging to determine which approach is the most beneficial. A comparative study on different mindfulness practices found that mindful yoga was associated with greater improvement in psychological well-being than sitting meditation and body scan techniques [24]^.^ However, further comparative studies are needed to identify the most effective mindfulness-based interventions tailored to different age groups.

Schools provide an ideal platform for delivering such interventions, as they offer structured environments to reach large adolescent populations during the formative stages of development. School-based yoga and mindfulness programs have demonstrated feasibility, acceptability, and effectiveness in promoting emotional well-being, reducing stress, and fostering adaptive coping skills among students [25,26]. In India, yoga is widely practiced in schools, often through standardized approaches such as the Common Yoga Protocol (CYP) developed by the Ministry of AYUSH. However, these practices are typically not integrated with structured mindfulness components [26]. Given the growing emphasis on mental health promotion within educational settings, there is a need to evaluate whether integrated approaches, such as Mindful Yoga and Meditation (MYM), offer additional benefits over standard yoga practices. Therefore, this study aimed to compare the effectiveness of MYM and CYP in enhancing emotion regulation and overall well-being among secondary school students.

The development of the MYM was grounded in the integration of Advancing Social and Emotional Learning (SEL) [27] and Mind-Body Connection Theory [28]. The SEL framework provides a systemic approach to cultivating core competencies, such as self-awareness, emotional regulation, and interpersonal relationship skills [27]. Complementing this, Mind-Body Connection Theory provides the physiological and psychological basis for the intervention, emphasizing bidirectional communication between the central nervous system and peripheral physiological processes. Within the MYM intervention, this theory posits that mindful movement and breathwork facilitate “bottom-up” regulation of the autonomic nervous system, which in turn enhances “top-down” cognitive control [28].

## Method

### Research design

The study employed a three-arm quasi-experimental design to evaluate the effectiveness of MYM and Common Yoga Protocol (CYP) interventions on psychological distress, emotion regulation, mindfulness, and well-being among school-going adolescents. Two parallel-group trials were conducted, and a waitlist control group continued with the standard school curriculum without additional interventions.

### Study setting

The study was conducted in two secondary schools affiliated with the Karnataka State Board in the Udupi district, located in the coastal region of southern Karnataka, India. According to the 2011 Census, Udupi is a prominent district with a population of 1, 177, 361, comprising 562, 131 males and 615, 230 females. Of this population, 926,429 individuals were reported to be literate (465,704 males and 460,725 females), resulting in an overall literacy rate of 86%, which is significantly higher than the national average of 59.5%. The male and female literacy rates are 91% and 82%, respectively, with females accounting for 52% of the total population [29,30].

According to the State Education Board of Karnataka, the district is divided into five educational blocks: Udupi, Brahmavara, Byndoor, Karkala, and Kundapura, encompassing a total of 249 secondary schools [31]. For this study, schools located in the Udupi and Kundapura blocks were selected as the primary sites of data collection.

### Participants

**Fig 1:**
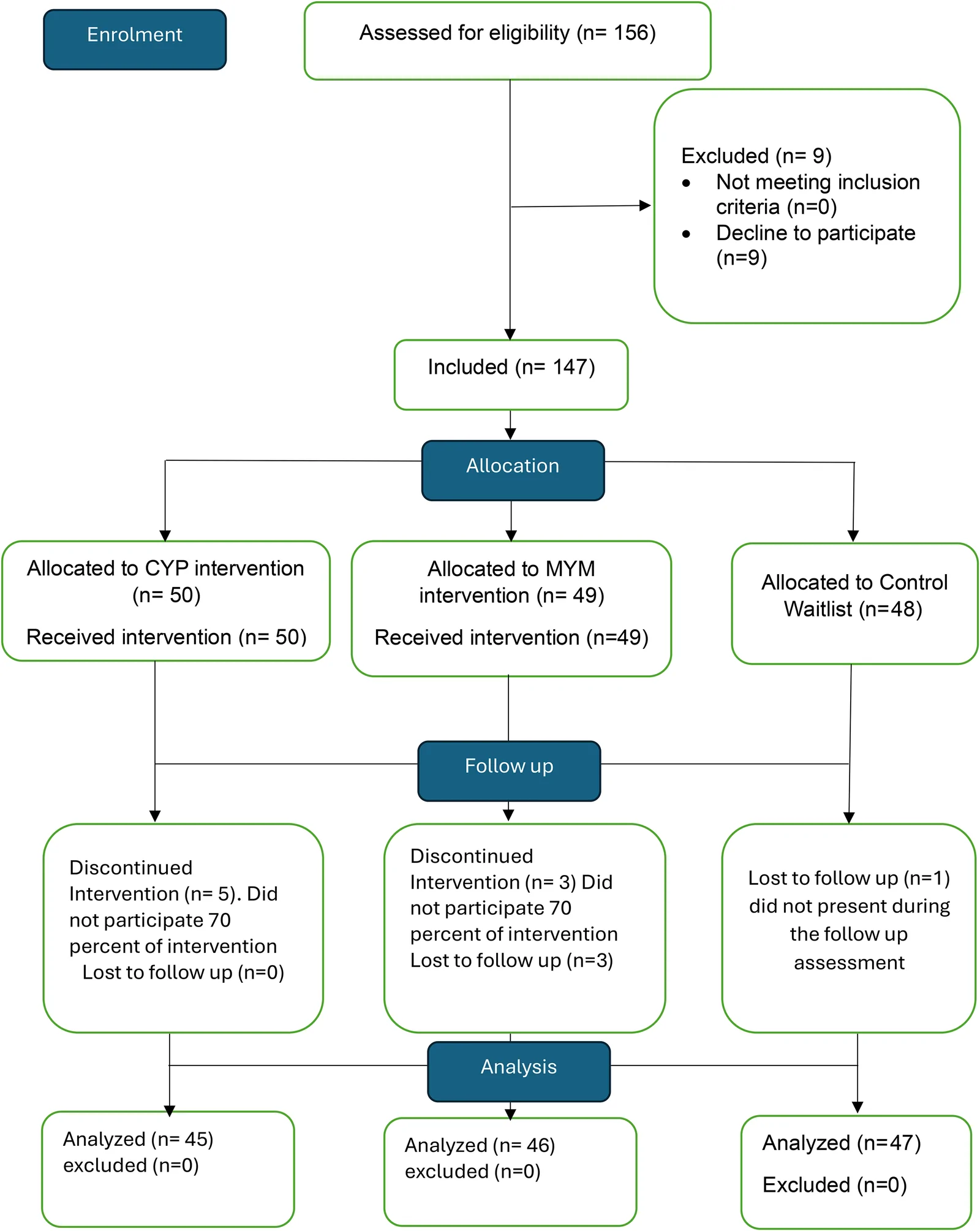
CONSORT flow diagram.

The participants in the study were secondary school students aged between 13 and 17 years (N = 147), recruited from two schools situated in the Udupi district of Karnataka, India. Allocation was determined by school-level feasibility and logistical considerations rather than random assignment. One school contributed participants to the CYP group, whereas the second school provided participants for the MYM and waitlist-control groups. The two schools were 40 km apart, which helped minimize contamination between the intervention arms (Fig 1).

### Sample size estimation

Based on the sample size calculation at a 5% level of significance with 80% power and a medium effect size (Cohen’s f = 0.25), the minimum sample size (per group) required for a three-group repeated measures study design involving quantitative outcomes (psychological distress, mindfulness, emotion regulation and well-being) measured across three time-points [pre, post and after 3-months] (correlation among repeated measures is 0.3), for a non-randomized control trial for the mindfulness-base yoga intervention among adolescents (Class 8–10) in Udupi district of Karnataka is 87. Incorporating a dropout rate of 10% and a design effect (d = 1.5), the minimum sample size requirement is 147, that is, 49 per group.


$$\begin{array}{c} n=\frac{\text{2}}{\delta^{\text{2}}}\left[\sigma_{b}^{\text{2}}+\;\frac{\sigma^{\text{2}}}{k}\left[\text{1}+(k-\text{1})\rho \right]\right]{\left(\left(Z_{\text{1}-\frac{\alpha }{\text{2}}}\right)+\left(Z_{\text{1}-\beta }\right)\right)}^{\text{2}} \end{array}$$

where, $\sigma_{b}^{\text{2}}$: between group variance, $\sigma^{\text{2}}$: within group variance, ρ: intra-class correlation coefficient, δ: effect size, k: number of repeated measures, α: level of significance and 1−β: power.

### Intervention

Both the MYM and CYP interventions were delivered by the principal investigator who had expertise in mindfulness and yoga.

#### MYM intervention.

The MYM intervention was developed through a systematic, multiphase iterative process designed to ensure theoretical soundness and stakeholder acceptability. A comprehensive literature review was conducted to identify the core evidence-based components of mindfulness and yoga that are effective for the adolescent demographic. Next, to ensure contextual relevance, qualitative data were gathered through Focus Group Discussions (FGDs) and In-Depth Interviews (IDIs) with a diverse stakeholder group, including secondary school students, parents, teachers, and mental health professionals [31]. Subsequently, a preliminary intervention draft was developed by synthesizing the findings from Phases I and II. This content underwent formal content validation by a panel of experts. The validated intervention was pilot-tested with a sample of adolescents to evaluate feasibility and refine delivery protocols prior to full-scale implementation. The MYM intervention module consists of three structured lesson plans [31] (see Table 1). The first lesson introduced the concepts of yoga and mindfulness, highlighting their benefits, and included the practice of fundamental yoga postures. The second lesson builds on this foundation by exploring the basic functions of the nervous system, the impact of stress, and how meditation influences various brain systems. This session incorporated basic yoga postures, breath awareness, and a short body scan meditation. The third lesson emphasizes the significance of cultivating compassion and gratitude by integrating mindful yoga postures with loving-kindness and gratitude meditation practices.

**Table 1:** Details of the MYM and CYP intervention.

Week	Mindful Yoga & Meditation (MYM)	Common Yoga Protocol (CYP)
1	The program focused on introducing the basic concepts of yoga and mindfulness, their practice methods, and associated benefits, alongside basic yoga postures.	Introduction to yoga practice and its benefits. The participants were guided through foundational yoga postures (āsana), which were practiced throughout the first week.
2	Introduction to the basic functions of the nervous system, the impact of stress, and how meditation influences various brain systems. The sessions included mindful yoga postures, awareness of breathing, and short body scan meditation practices.	In addition to yoga postures, basic breathing exercises (Prāṇāyāma) were introduced to enhance breath awareness and regulation
3	The importance of cultivating compassion and gratitude, along with the practical application of mindfulness in daily life, was emphasized. The participants engaged in mindful yoga postures, loving-kindness, and gratitude meditation practices.	Meditation (Dhyāna) was incorporated alongside yoga postures and breathing exercises, allowing the participants to deepen their practice and cultivate mindfulness.

The intervention was conducted in the school auditorium, a spacious, well-ventilated, and clean environment conducive to mindfulness and yoga. A total of 49 students participated in the MYM intervention. Each participant was provided with a yoga mat and instructed to use it consistently during the program. The sessions were scheduled one hour before the regular classes, each lasting for 45 minutes. The first session focused on building rapport and establishing ground rules to foster a supportive and engaging learning environment for the students. Ice-breaking activities were incorporated to encourage participant interaction, followed by a detailed program overview to ensure clarity and alignment with its objectives.

On the second day, the participants were introduced to the foundational concepts of yoga and mindfulness through an interactive face-to-face session supported by a 20-minute PowerPoint presentation. This was followed by a live demonstration of simple yoga postures, which allowed the participants to engage in hands-on learning. The intervention was structured over three weeks. Details of the intervention are provided in Table 1.

By the end of the program, the participants were provided with detailed instructions on continuing their practice at home or school. A structured home practice guide was distributed to support independent practice. Feedback and suggestions were collected, and participants were encouraged to maintain a daily diary to document their experiences, challenges, and progress in mindfulness practice. A post-test assessment was conducted to evaluate the effectiveness of the intervention. Of the 49 participants, 46 completed the intervention and post-test assessments, while three participants dropped out. They were encouraged to sustain their practice for three months. To assess the long-term impact of the intervention, all tests were repeated after three months, providing insights into its effects.

#### CYP intervention.

The CYP intervention adhered to traditional yoga practices based on the Common Yoga Protocol (CYP) developed by the Ministry of AYUSH, India [32]. The protocol incorporated a structured sequence of yoga postures (āsana), breathing exercises (prāṇāyāma), and meditation (dhyāna) to promote overall well-being.

The intervention was conducted in a well-ventilated and clean school auditorium, providing an ideal setting for practice. A total of 50 students enrolled in the CYP group participated in a three-week yoga intervention, attending 45-minute daily sessions. Of the 50 participants, 45 completed the intervention, while five dropped out.

All sessions were conducted face-to-face and featured live demonstrations to ensure proper technique and alignment. A post-test was conducted to assess the psychological distress, emotion regulation, mindfulness, and well-being of the participants. The participants were encouraged to maintain their yoga practice for the next three months. Home practice guidelines were distributed to the participants. To assess the long-term impact of the intervention, all assessments were repeated after three months, providing insights into sustained benefits over time.

### Data collection tools

#### Socio-demographic Data Sheet.

Socio-demographic Data Sheet was prepared by the researcher for the current study to collect socio-demographic details such as age, class, gender, religion, family type, education of the parents, occupation of the parents, siblings and socio economic status of the parents (see Table 2).

**Table 2:** Socio-demographic characteristics of the study population at baseline.

Demographic characteristics		Control (n = 47) n(%)	CYP (n = 45) n(%)	MYM (n = 46) n(%)	p-value
Gender	Male	24 (51.1)	15 (33.3)	27 (58.7)	0.046*
	Female	23 (48.9)	30 (66.7)	19 (41.3)	
Class	8	0	0	18 (39.1)	<.001*
	9	16 (34)	0	28 (60.9)	
	10	31 (66)	45 (100)	0	
Religion	Hindu	39 (83)	43 (95.6)	41 (89.1)	0.153
	Other	8 (17)	2 (4.4)	5 (10.9)	
Family Type	Nuclear	28 (59.6)	41 (91.1)	27 (58.7)	<.001*
	Joint	19 (40.4)	4 (8.9)	19 (41.3)	
Education of Mother	School Education	21 (51.2)	23 (69.7)	7 (28)	0.009*
	Pre-University	11 (26.8)	5 (15.2)	14 (56)	
	Graduate and above	9 (22)	5 (15.2)	4 (16)	
Education of father	School Education	24 (57.1)	30 (83.3)	5 (20)	<.001*
	Pre-University	4 (9.5)	4 (11.1)	10 (40)	
	Graduate and above	14 (33.3)	2 (5.6)	10 (40)	
Occupation of Mother	Employed	8 (17)	28 (71.8)	5 (10.9)	<.001*
	Not employed	36 (76.6)	9 (23.1)	40 (87)	
	Self employed	3 (6.4)	2 (5.1)	1 (2.2)	
Occupation of Father	Employed	36 (76.6)	8 (21.1)	31 (68.9)	<.001*
	Self employed	11 (23.4)	30 (78.9)	14 (31.1)	
Siblings	No sibling	7 (61.3)	0	9 (19.6)	<.001*
	One sibling	36 (83.7)	26 (66.7)	34 (73.9)	
	More than one sibling	0	13 (33.3)	3 (6.5)	
Socio Economic Status	Middle Class	46 (97.9)	42 (93.3)	45 (97.8)	0.412
	Upper Class	1 (2.1)	3 (6.7)	1 (2.2)	
Age (Years)		Mean ±SD	Mean ±SD	Mean ±SD	<.001*
		15 (0.69)	15.1 (0.57)	13.7 (0.57)	

*significant at 5% level of significance.

#### Kessler psychological distress scale (K10).

The K10 is a freely available 10-item self-report questionnaire designed to measure non-specific psychological distress, focusing on symptoms of anxiety and depression experienced over the past four weeks [33]. Each item is rated on a 5-point Likert scale ranging from “None of the time” (1) to “All of the time” (5), yielding a total score between 10 and 50, with higher scores indicating greater distress. The K10 has demonstrated high internal consistency, with Cronbach’s alpha values typically exceeding 0.90.

#### Emotion regulation questionnaire for children and adolescents (ERQ-CA).

The ERQ-CA is a 10-item self-report instrument adapted from the adult version of the Emotion Regulation Questionnaire (free to use with author permission) [34]. It assesses two emotion regulation strategies: Cognitive Reappraisal (six items) and Expressive Suppression (four items). Responses were recorded on a 5-point Likert scale ranging from “Strongly disagree” (1) to “Strongly agree” (5). The ERQ-CA has demonstrated good internal consistency, with Cronbach’s alpha values of 0.79 for Cognitive Reappraisal and 0.73 for Expressive Suppression.

#### Children and adolescent mindfulness measure (CAMM).

The CAMM is a 10-item self-report measure designed to assess mindfulness in children and adolescents, particularly focusing on present-moment awareness and non-judgmental acceptance of thoughts and feelings [35]. Items are rated on a 5-point Likert scale from “Never true” (0) to “Always true” (4), with higher scores indicating greater mindfulness. The CAMM has shown satisfactory internal consistency, with Cronbach’s alpha values ranging from 0.71 to 0.80 across different age groups.

#### WHO-5 well-being index.

The WHO-5is a brief, 5-item, self-report, freely available questionnaire assessing subjective psychological well-being over the past two weeks [36]. Each item is rated on a 6-point Likert scale from “At no time” (0) to “All of the time” (5), resulting in a total score ranging from 0 to 25, with higher scores indicating better well-being. WHO-5 has demonstrated high internal consistency, with Cronbach’s alpha values typically above 0.80.

### Researcher positionality

The researcher’s positionality was informed by their prior experience in yoga and mindfulness training. The researcher completed a master’s program in Yoga Therapy and holds a certification in mindfulness life coaching. While this background facilitated rapport and contextual understanding, conscious efforts were undertaken to minimize potential bias through the use of standardized measures, structured intervention protocols and supervision throughout the research process.

### Data analysis

Data were analyzed using Jamovi [37]. The four psychological outcome variables were summarized using descriptive statistics, including the mean, standard deviation, median, and interquartile range. Descriptive statistics, including the mean, standard deviation, frequency, and percentages, were used to describe the sociodemographic characteristics of the study participants.

A linear mixed-effects model (LMM) using a restricted maximum likelihood (REML) approach with bobyqa optimization was employed to examine the effectiveness of MYM,CYP, and a waitlist control group on psychological distress (K10), mindfulness (CAMM), emotion regulation (ERQ-CR and ERQ-ES), and well-being (WHO-5) across three time points. The LMM was selected because of its suitability for analyzing repeated measures data, as it accounts for the non-independence of observations within individuals over time and allows for the inclusion of participants with incomplete data without requiring listwise deletion.

The model included fixed effects for group, time, and group × time interaction, with participants treated as random effects to account for the individual variability. This approach enables a robust estimation of intervention effects while accommodating intra-individual correlation. The LMM inherently adjusts for baseline differences by modeling all time points simultaneously and estimating changes from baseline within each group, rather than relying solely on post-intervention comparisons. The inclusion of baseline measurements as part of the repeated-measures structure ensured that any pre-existing differences between groups were accounted for in the estimation of intervention effects.

### Ethical consideration and approvals

All participants were thoroughly briefed on the aims and objectives of the study and the nature and scope of their involvement. They were informed of their rights as research participants, including the right to withdraw from the study at any stage without negative consequences. Participation was entirely voluntary, and it was emphasized that refusal to participate would not impact the participants in any way. Participants who scored severe distress range on the K10 were referred to the school authorities to ensure appropriate professional mental health support.

Written informed assent was obtained from all student participants, along with written informed consent from their parents or legal guardians. Confidentiality and anonymity were strictly maintained throughout the study period. The researcher duly acknowledged all the research instruments used in the study.

The study secures all necessary permissions and approvals. Ethical approval was obtained from the Institutional Ethics Committee of Kasturba Hospital and Kasturba Medical College, Manipal, MAHE [Reg No. ECR/146/Inst/KA/2013/RR-19; Ref: IEC1–295–2022] (Refer S). The study was registered with the Clinical Trial Registry of India [CTRI/2023/06/053499; registered on 02/06/2023] (Refer S). Permission from the Deputy Director of Public Instruction (DDPI) Udupi district, Karnataka, and from the school authorities was obtained before the implementation of the study.

## Results

Data analysis was performed to compare the effectiveness of Mindful Yoga and Meditation (MYM) and Common Yoga Protocol (CYP) on psychological distress (K10), mindfulness (CAMM), emotion regulation (ERQ-CR and ERQ-ES), and well-being (WHO-5) in adolescent students using Jamovi 2.3.28, a free graphical user interface for R programming language (The jamovi project, 2024).

### Baseline comparison of demographic details between groups

Socio-demographic characteristics varied across the three groups (Table 2). Significant differences were observed in gender distribution (p = 0.046), class (p < .001), family type (p < .001), parental education (mothers: p = 0.009; fathers: p < .001), parental employment (p < .001), number of siblings (p < .001), and age (p < .001). The CYP group had a higher proportion of females and consisted entirely of class 10 students, whereas the MYM group included younger students from classes 8 and 9. Family structure and parental characteristics also differed, with the CYP group showing higher rates of nuclear families, employed mothers, and self-employed fathers than the non-CYP group. No significant differences were found in religion (p = 0.153) or socioeconomic status (p = 0.412), with most participants identifying as Hindu and belonging to the middle class.

### Comparison of the effectiveness of MYM and CYP intervention over three time period

**Fig 2:**
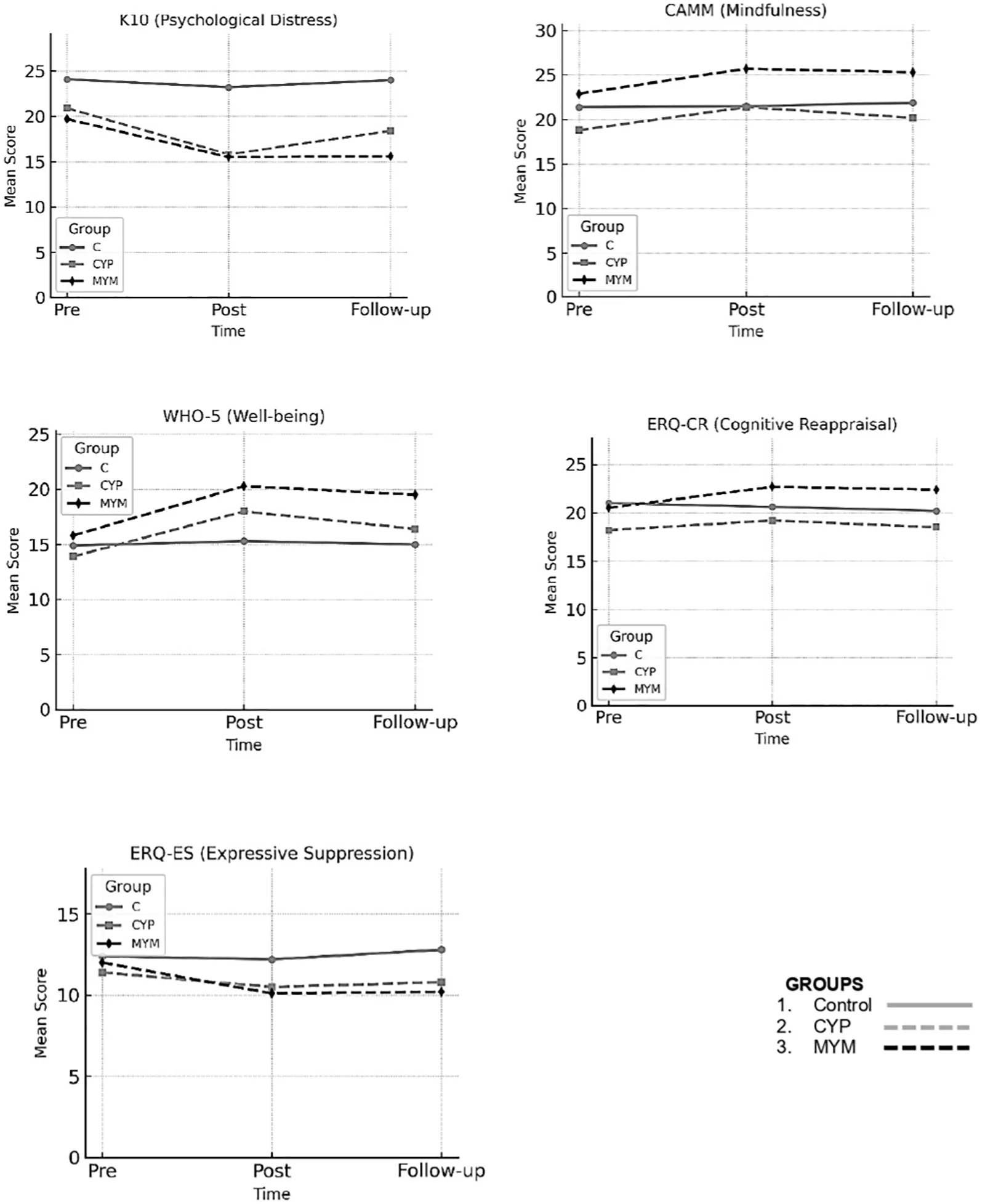
Profile plots comparing MYM and CYP effects on K10, CAMM, ERQ-CA, and WHO-5 scores across three time points.

A linear mixed-effects model (LMM) using the restricted maximum likelihood approach with bobyqa optimization was conducted to examine the effects of two interventions, CYP and MYM, along with the waitlist control on four outcomes: psychological distress, mindfulness, emotion regulation, and well-being, assessed over three time points. The model included group (CYP, MYM, Control), time (T1, T2, T3), and their interaction (Group × Time) as fixed effects, with participant ID as a random effect to account for individual variability. Table 3 presents the results of the LMM analysis. Fig 2 presents a graphical representation of the comparisons between groups over time. The Bonferroni-Adjusted post hoc Pairwise Comparisons are given as supplementary material (S1 File).

**Table 3:** Comparison of the effectiveness of MYM and CYP intervention on psychological distress (K10), mindfulness (CAMM), emotion regulation (ERQ-CR, ES), and well-being (WHO-5) over three-time period.

Outcome	Group	Pre-Assessment	Post-Assessment	Follow-up	Source of variation	Linear Mixed Model: Analysis		
		Mean±SD	Mean±SD	Mean±SD		F (df1, df2)	p-value	Effect Size
K10	Control	24.1 ± 4.85	23.2 ± 4.08	24 ± 4.3	Time	53.4 (2, 270)	< .0001*	0.283
	CYP	20.9 ± 6.39	15.8 ± 4.26	18.4 ± 4.05	Group	38.2 (2,135)	< .0001*	0.131
	MYM	19.7 ± 5.38	15.5 ± 3.9	15.6 ± 3.45	Time*Group	10.2 (4,270)	< .0001*	0.361
CAMM	Control	21.4 ± 5	21.5 ± 5.05	21.9 ± 4.79	Time	19.12 (2, 270)	< .0001*	0.124
	CYP	18.8 ± 4.3	21.4 ± 4.92	20.2 ± 4.15	Group	13.7 (2,135)	< .0001*	0.06
	MYM	22.9 ± 5.35	25.7 ± 4.6	25.3 ± 4.14	Time*Group	4.34 (4,270)	0.002*	0.169
ERQ-CR	Control	21 ± 2.92	20.6 ± 3.13	20.2 ± 2.76	Time	11.7 (2, 270)	< .0001*	0.08
	CYP	18.2 ± 2.8	19.2 ± 2.78	18.5 ± 2.64	Group	16.8 (2, 135)	< .0001*	0.151
	MYM	20.5 ± 3.96	22.7 ± 2.84	22.4 ± 2.45	Time*Group	12 (4,270)	< .0001*	0.199
ERQ-ES	Control	12.4 ± 2.64	12.2 ± 2.81	12.8 ± 2.9	Time	17.77 (2, 270)	< .001*	0.116
	CYP	11.4 ± 2.04	10.5 ± 1.71	10.8 ± 1.66	Group	9.49 (2,135)	< .001*	0.099
	MYM	12 ± 3.04	10.1 ± 2.6	10.2 ± 2.25	Time*Group	7.44 (4, 270)	< .001*	0.123
WHO-5	Control	14.9 ± 3.59	15.3 ± 3.55	15 ± 3.63	Time	41.69 (2, 270)	< .0001*	0.236
	CYP	13.9 ± 4.56	18 ± 3.8	16.4 ± 3.18	Group	17.5 (2,135)	< .0001*	0.116
	MYM	15.8 ± 4.72	20.3 ± 2.87	19.5 ± 2.57	Time*Group	8.88 (4,270)	< .0001*	0.206

*significant at 5% level of significance.

#### Psychological distress.

The model fit for the LMM model statistics for K10 indicated strong explanatory power, with a marginal R² of 35.4%, which indicates the variance explained by fixed effects, and a conditional R² of 76.5%, which indicates the variance explained by both fixed and random effects. A significant main effect of group (F (2,135) = 38.2, p < .0001) and time (F (2,270) = 53.4, p < .0001) was found, indicating that distress levels varied significantly between groups and changed over time. The significant Group × Time interaction (F (4,270) =10.2, p < .0001) suggests that distress reduction differed across groups, supporting the effectiveness of the interventions. Post hoc comparisons using Bonferroni tests revealed that both the CYP and MYM groups had significantly lower distress scores than the control group at all time points (p < .0001). Specifically, at baseline, participants in the CYP (p < .0001) and MYM (p < .0001) groups reported significantly lower distress scores than those in the control group. Over time, distress levels significantly decreased across all groups, with the largest reductions observed between T1 and T2 (p < .0001). The Group × Time interaction effects indicated that the CYP and MYM groups showed significantly greater reductions in distress than the control group, particularly at T2 (p < .0001). Furthermore, an assessment of the K10 scores at each of the three time points revealed the following:

At T1, both intervention groups reported significantly lower K10 scores than the control group. The CYP group differed significantly from the control group (p < .0001), while the MYM group reported significantly lower distress than the control group (p < .0001). There was also a significant difference between CYP and MYM at T1 (p = .0204), suggesting that MYM participants initially experienced less distress than CYP. At T2, the pattern of group differences persisted, with CYP continuing to show significantly lower distress scores than the control group (p < .0001), and MYM participants also reported less distress than the control group (p < .0001). The difference between CYP and MYM was not statistically significant (p > 0.99), indicating a convergence in symptom reduction between the two interventions at this point. By T3, both intervention groups maintained significantly lower K10 scores than the control group. However, there was again no significant difference between CYP and MYM at this time point (p = .159), suggesting sustained effects of both interventions, although the magnitude of distress appeared greater for CYP.

Within-group changes over time provide further insights. The CYP group showed large and statistically significant reductions in distress from T1 to T2 (p < .0001) and from T1 to T3 (p < .0001), with no significant change between T2 and T3, suggesting the stability of intervention effects. Similarly, the MYM group exhibited significant reductions from T1 to T2 (p < .0001) and from T1 to T3 (p = .0008), with a non-significant change between T2 and T3 (p = .159), again indicating the sustainability of the intervention in reducing psychological distress. In contrast, the control group did not show significant differences between any time points: T1 vs. T2 (p > 0.99), T1 vs. T3 (p = .2204), and T2 vs. T3 (p > 0.99), highlighting the absence of spontaneous recovery in psychological distress without intervention.

Taken together, these findings provide robust evidence for the effectiveness of both CYP and MYM interventions in reducing psychological distress over time, with the MYM group exhibiting the most consistent and substantial improvement over time. However, no significant difference was found between the CYP and MYM groups (p = .257), suggesting that both interventions were equally effective in reducing distress. The random effects analysis revealed that 63.5% of the variance in distress levels was attributable to individual differences, supporting the inclusion of participant-level random intercepts in the model. The significant interaction effects between group and time suggest that the interventions contributed to distress reduction beyond the natural fluctuations over time. Overall, these findings indicate that both CYP and MYM interventions effectively reduce psychological distress, with improvements being maintained over time.

#### Mindfulness.

The model fit for the linear mixed model (LMM) analyzing CAMM scores indicated moderate explanatory power, with a marginal R² of 16.6%, reflecting the variance explained by fixed effects, and a conditional R² of 73.7%, reflecting the variance explained by both fixed and random effects. A significant main effect of group (F(2,135) = 13.7, p < .0001) and time (F(2,270) = 19.12, p < .0001), and a significant Group × Time interaction (F(4,270) = 4.34, p = .002) was observed, indicating that mindfulness scores varied across groups and time, and that the pattern of change differed by group.

Post hoc comparisons using Bonferroni correction showed that, at baseline, MYM participants scored significantly higher in mindfulness than the control group (p = .002), while the difference between the CYP and control groups was not statistically significant (p = .306). Over time, all groups showed increases in CAMM scores, with the most notable improvements occurring between T1 and T2 (p < .0001). Group × Time interaction effects indicated that both intervention groups demonstrated greater increases in mindfulness than the control group, particularly at T2.

At T1, the MYM group exhibited significantly higher mindfulness than both the control and CYP groups (p = .002 and p < .0001, respectively), indicating a strong initial effect of mindful yoga training. At T2, both the MYM and CYP groups showed significantly elevated CAMM scores relative to the control group (p < .0001 for both), although the difference between the MYM and CYP groups was not statistically significant (p = 1.000). This suggests that CYP experienced a delayed gain in mindfulness compared to MYM. By T3, MYM participants maintained significantly higher mindfulness than the control group (p = .017), while the CYP group no longer differed significantly from the control group (p > 0.99), suggesting that mindfulness improvements persisted for MYM but not for CYP.

Within-group comparisons over time showed that MYM participants experienced significant increases in mindfulness from T1 to T2 (p < .001) and from T1 to T3 (p < .001), with no significant change between T2 and T3 (p > 0.99), indicating that the improvements were sustained. The CYP group also showed significant gains from T1 to T2 (p < .001) and T1 to T3 (p = .001), but with a decline noted by T3. In contrast, the control group exhibited no significant changes across time points (p > .05 for all), underscoring the absence of natural changes in mindfulness without intervention.

These findings suggest that both interventions improved mindfulness, but the MYM module produced stronger and more sustained gains. The random effects analysis revealed that 68.4% of the variance in CAMM scores was attributable to individual differences, further justifying the inclusion of participant-level random intercepts in the model.

#### Emotional regulation – Cognitive reappraisal.

The LMM for ERQ-CR scores demonstrated moderate explanatory power, with a marginal R² of 19.8% and a conditional R² of 78.6%, indicating a substantial contribution from both fixed and random effects. Significant main effects of group (F(2,135) = 16.8, p < .0001) and time (F(2,270) = 11.7, p < .0001) and a significant Group × Time interaction (F(4,270) = 12.0, p < .0001) were observed.

Post hoc comparisons revealed that at baseline, the MYM group scored significantly higher in cognitive reappraisal than the control group (p = .026), whereas the CYP group scored significantly lower than the control group (p < .001). Over time, reappraisal scores increased across all groups, with the most substantial changes observed between T1 and T2 (p < .001). The Group × Time interaction showed that both the CYP and MYM groups experienced significantly greater increases in reappraisal than the control group at T2 and T3.

At T1, MYM participants had significantly higher ERQ-CR scores than the CYP group (p = .002), suggesting a stronger baseline regulatory capacity. By T2, both intervention groups showed significant improvements compared to the control group (CYP: p = .001; MYM: p < .001), and these improvements were maintained at T3 (CYP: p = .017; MYM: p < .001). The MYM group consistently showed the highest scores, although the differences between the CYP and MYM groups at follow-up were not significant (p > 0.99), suggesting convergence in long-term outcomes.

Within-group analyses showed that MYM participants had significant gains from T1 to T2 (p < .001) and from T1 to T3 (p = .001), with no significant change between T2 and T3, indicating lasting effects of the intervention. CYP participants also improved from T1 to T2 (p < .001) and from T1 to T3 (p = .023), with stable outcomes. The control group exhibited no significant changes in the time points.

Together, these results support the effectiveness of both interventions, particularly MYM, in enhancing adolescents’ cognitive reappraisal skills. The ICC of 73.4% indicates that a substantial portion of the variability was due to individual differences, underscoring the robustness of the intervention effects.

#### Emotional regulation – Expressive suppression.

The linear mixed model (LMM) for the ERQ-ES revealed modest explanatory power, with a marginal R² of 13.9%, indicating the variance explained by fixed effects, and a conditional R² of 68.6%, reflecting the total variance explained by both fixed and random effects. A significant main effect was found for Group (F(2,135) = 9.49, p < .001), Time (F(2,270) = 17.77, p < .001), and Group × Time interaction (F(4,270) = 7.44, p < .001), indicating that expressive suppression scores changed significantly over time and that patterns of change differed between groups.

Post hoc comparisons using Bonferroni correction revealed that both CYP (p = .002) and MYM (p < .001) had significantly lower ERQ-ES scores than the control group when averaged across time points. However, the difference between CYP and MYM was not significant (p = 1.000), suggesting that the two interventions had similar average levels of suppression.

Over time, suppression scores significantly decreased across all groups, with the most notable reduction occurring between T1 and T2 (p < .001) and a smaller yet significant decrease between T1 and T3 (p < .001). The decrease between T2 and T3 was not significant (p = .188), indicating that most changes occurred during the intervention period and then stabilized. At T1 (baseline), there were no significant differences between the groups in suppression scores (all p > .05), although the CYP group had slightly lower values than the control group. By T2, both intervention groups showed significantly lower ERQ-ES scores than the control group (CYP: p = .007; MYM: p < .001), with the MYM group showing the greatest reduction. This trend continued at T3, with the MYM and CYP groups maintaining significantly lower scores than the control group (MYM: p < .001; CYP: p = .004), suggesting sustained reductions in expressive suppression. However, there was no significant difference between CYP and MYM at follow-up’.

Within-group comparisons revealed that MYM participants showed significant reductions in suppression from T1 to T2 (p < .001) and from T1 to T3 (p < .001), with no significant change between T2 and T3 (p > 0.99), indicating stable post-intervention effects. A similar pattern was observed in the CYP group, with significant decreases from T1 to T2 (p < .001) and marginal changes thereafter. In contrast, the control group showed no significant within-group changes over time, underscoring the role of the interventions in driving improvements.

The random effects analysis indicated that 63.6% of the variance in expressive suppression was attributable to individual differences, further supporting the use of participant-level random-effects intercepts. These results suggest that both yoga-based interventions effectively reduced adolescents’ reliance on expressive suppression, with the MYM program showing slightly more consistent and robust effects.

#### Well-being.

The LMM analysis of the WHO-5 well-being scores showed a good model fit, with a marginal R² of 24.3% and a conditional R² of 56.3%, suggesting that both fixed and random effects contributed to the variance in well-being. A significant main effect of group (F(2,135) = 17.5, p < .0001), time (F(2,270) = 41.69, p < .0001), and group × time interaction (F(4,270) = 8.88, p < .0001) were found.

Post-hoc tests revealed that MYM participants reported significantly higher well-being than the control group at baseline (p < .001), while the difference between CYP and the control was not statistically significant (p = .250). Over time, both the CYP and MYM groups showed significant improvements in well-being, especially between T1 and T2 (p < .001), with sustained benefits at T3. The Group × Time interaction indicated that these improvements were significantly greater than those in the control group. At T2, both intervention groups had significantly higher WHO-5 scores than the control group (CYP: p = .002; MYM: p < .001), and this pattern remained consistent at T3 (CYP: p = .005; MYM: p < .001). There was no significant difference between CYP and MYM at either the post- or follow-up time points (p = .152), although the magnitude of improvement was greater in the MYM group.

Within-group comparisons indicated significant improvements in both intervention groups from T1 to T2 and T1 to T3 (p < .001), with no significant changes between T2 and T3, suggesting sustained benefits. In contrast, the control group showed no significant change in well-being across any time point (p > .05), indicating that the observed improvements were attributable to the interventions.

These findings underscore the efficacy of both yoga-based interventions in enhancing adolescent well-being, with MYM producing slightly more pronounced effects. Random intercept analysis showed an ICC of 42.3%, reflecting considerable individual variability in well-being levels across the participants.

## Discussion

This study examined the comparative effectiveness of two yoga-based interventions, mindful Yoga and Meditation (MYM) and the Common Yoga Protocol (CYP), in reducing psychological distress and enhancing mindfulness, emotion regulation, and well-being among adolescents. By doing so, this study addresses a critical gap in adolescent-specific mindfulness interventions, showcasing how a structured, activity-based module such as the MYM intervention can significantly enhance well-being, emotion regulation, and mindfulness among school-going adolescents. While most mindfulness-based programs for adolescents are adopted from mindfulness-based stress reduction (MBSR) [38] or mindfulness programs for adults, the MYM module is uniquely tailored to adolescents’ developmental needs and preferences, incorporating engaging, activity-based elements that resonate well with this age group. Recognizing the need for contextually appropriate interventions, this study leverages formative research to explore the specific needs, barriers, and facilitators of incorporating mindfulness and yoga practices within the Indian school setting [31].

### Development of a contextually relevant intervention

The study was conducted in three distinct phases (see ref 31). The first phase involved formative research using qualitative methods to explore the perceived needs, barriers, and support systems for implementing mindfulness-based yoga practices in school settings. Insights from this phase informed the development of a contextually relevant intervention. In the second phase, a structured mindfulness-based yoga and meditation module tailored for adolescents (MYM) was developed and piloted for feasibility and relevance. The final phase employed a quasi-experimental design to evaluate the effectiveness of the MYM intervention compared with an existing traditional Hatha yoga-based protocol (CYP). This multiphase approach ensured that the intervention was both evidence-informed and contextually grounded, enabling a comprehensive evaluation of its impact on adolescent mental health outcomes.

The formative research findings underline the need to integrate yoga and mindfulness practices in school settings in India to address the growing concerns regarding adolescent mental health. Both teachers and parents recognized the potential of these practices in promoting emotional regulation, stress management, and improved focus among students. However, the integration of these practices faces several barriers, including academic pressure, time constraints, a lack of trained professionals, and societal stigma. The findings also highlight several facilitators that could help overcome these challenges, such as increasing awareness, integrating yoga into existing programs, gaining support from school administrations, and involving parents in the process. These facilitators are crucial for creating a supportive environment in which yoga and mindfulness practices can be effectively integrated into the school curricula.

The MYM module was meticulously designed to help adolescents cultivate mindfulness and yoga practices over 21 days, with each session lasting 45 minutes. This timeframe was intentionally chosen to encourage habit formation and ensure that the participants developed proficiency in the practices. Previous studies have shown that the consistency and duration of mindfulness-based practices play crucial roles in determining the extent of positive outcomes. Regular and sustained engagement with these practices has been linked to greater improvements in mental health and overall well-being [20,39,40]. Long-term mindfulness programs are required to sustain mental health and well-being among adolescents, rather than focusing on short-term interventions [21,40,41].

This program combines mindfulness techniques with yoga postures, offering a holistic approach to addressing common challenges faced by adolescents, such as emotional regulation difficulties, impulsivity, and poor attention spans. The activity-based format of the intervention ensures greater engagement and acceptability among the target group, distinguishing it from traditional mindfulness programs. The structure and duration of the program were carefully designed to seamlessly integrate mindfulness practices into the participants’ daily lives, fostering long-term habits that promote mental health and well-being.

An inclusive multidisciplinary approach was adopted to develop and validate the MYM intervention module. Input was sought from experts in yoga, mental health, and education to ensure that the program was both evidence-based and contextually relevant. While prior studies have relied primarily on yoga and mental health experts, the inclusion of education professionals in this study acknowledges the unique challenges and opportunities presented by adolescent learners [42,43]. Insights from parents, teachers, and students further enriched the module, ensuring that it was well aligned with the lived experiences and expectations of all stakeholders.

### Effectiveness of MYM intervention and alignment with existing literature

Both the MYM and CYP interventions were effective in significantly reducing psychological distress and enhancing well-being, mindfulness, and emotion regulation among adolescents. Importantly, participants in both intervention groups demonstrated significantly greater improvements across all outcome domains than those in the control group, underscoring the effectiveness of yoga-based practices for adolescent mental health. While both interventions yielded positive outcomes, the MYM group consistently exhibited slightly greater improvements, particularly in mindfulness and cognitive reappraisal, suggesting the potential added benefit of integrating mindfulness components into yoga-based interventions.

The findings of the present study align closely with and extend the growing body of literature supporting mindfulness- and yoga-based interventions for adolescents. Consistent with prior research, the MYM intervention demonstrated significant improvements in emotion regulation, mindfulness, and psychological well-being while reducing emotional suppression and psychological distress. Several studies have reported that mindfulness-based interventions (MBIs) improve adolescents’ ability to regulate their emotions and manage stress [23,44,45]. The MYM module targets these areas, offering a holistic approach that blends mindfulness with physical activity, a combination that resonates particularly well with adolescents, as evidenced by previous studies [46–49].

The reduction in psychological distress observed in both the MYM and CYP groups highlights the potential of school-based yoga interventions for supporting adolescent mental health. The significant Group × Time interaction confirms that distress reduction was not merely a function of time but was specifically associated with participation in either intervention. While the positive effects of both interventions began to slightly diminish over a three-month period, participants in the MYM group were able to sustain their benefits to a greater extent than those in the CYP group. The sustained decrease in distress suggests that the interventions may have equipped the students with lasting coping strategies. Notably, neither intervention group showed significant deterioration between the post-test and follow-up, indicating durable effects beyond the intervention period. In contrast, the control group did not experience meaningful changes in distress at any time point, ruling out spontaneous remission as an explanation.

The MYM intervention produced robust and enduring increases in dispositional mindfulness, whereas gains in the CYP group were more modest and transient. This finding aligns with theoretical perspectives positing that mindfulness, as a metacognitive capacity, requires deliberate and structured cultivation to produce meaningful trait-level changes [50]. The inclusion of mindfulness psychoeducation and daily practice components in the MYM intervention may have deepened participants’ understanding and motivation, with many students reporting informal use of brief mindfulness practices between academic sessions. These findings reinforce the importance of both theoretical orientation and practice continuity in producing sustained mindfulness-related outcomes.

Both MYM and CYP participants showed significant increases in cognitive reappraisal and reductions in expressive suppression, two key dimensions of emotion regulation. Improvements in reappraisal were particularly pronounced in the MYM group and remained stable over time, consistent with the proposed mechanism by which mindfulness enhances emotional flexibility through increased awareness and de-centering. While CYP participants also showed improvements in reappraisal, these were comparatively delayed and less robust, possibly reflecting the absence of a reflective or metacognitive component in the intervention. Reductions in expressive suppression were maintained throughout the follow-up period in both intervention groups, suggesting durable shifts in maladaptive regulation tendencies. Although the between-group differences in suppression were not statistically significant, the MYM group showed earlier and more pronounced reductions, potentially reflecting the added value of present-moment awareness and emotional acceptance fostered through mindfulness practices.

Interestingly, the CYP group achieved outcomes comparable to the MYM group on several indicators despite the lack of explicit mindfulness instruction. This underscores the intrinsic value of traditional yoga practices in fostering emotional regulation and psychological well-being. These findings resonate with prior research suggesting that physical and breath-based yogic practices may implicitly cultivate mindfulness, even without direct training [11,51]. However, the greater sustainability of outcomes in the MYM group supports the proposition that explicitly structured mindfulness interventions confer long-term benefits by reinforcing both theoretical understanding and practical application.

Facilitator expertise and fidelity of delivery are critical to the effectiveness of the intervention [52,53]. In the present study, the MYM module was designed for implementation by school teachers, supported by a structured curriculum, digital resources and guided audio practices. This teacher-led model likely enhanced contextual relevance, engagement, and sustainability, consistent with prior findings suggesting that school-embedded delivery may be more effective than programs led by external facilitators. The structured integration of mindfulness principles into existing school routines, such as brief practices between classes, may further promote consistent engagement and long-term impact. To facilitate broader dissemination and scalability of the program, a dedicated trainer-training module for MYM will need to be developed.

From a practical perspective, the findings suggest that school-based mindfulness and yoga interventions can be integrated into existing educational systems with relatively low resource requirements. The structured design of the MYM module, supported by guided audio practices and teacher-led delivery, enhances its feasibility and sustainability. Brief mindfulness exercises may be incorporated into classroom routines, morning assemblies, or transition periods between academic sessions. Such integration may provide a scalable and cost-effective strategy for promoting adolescent mental health in school settings.

The MYM intervention, with its structured and integrative design, demonstrated a slight yet meaningful advantage over traditional yoga in fostering sustained improvements in psychological functioning and emotion regulation. These results contribute to the growing body of literature supporting the inclusion of mindfulness-based practices in educational settings and highlight the potential of integrating such programs into school curricula to foster long-term mental health benefits among youth. Together, these findings provide preliminary evidence supporting the feasibility, acceptability, and potential effectiveness of brief school-based yoga interventions, particularly those incorporating explicit mindfulness components, in promoting adolescent mental health. Although the observed benefits are encouraging, larger randomized studies are needed to confirm these findings.

## Limitations

Despite these promising findings, this study has several limitations that should be acknowledged. First, the sample was drawn from two schools, which may affect the generalizability of the results to a broader population. Future studies should consider larger and more diverse samples to enhance their external validity. Second, while the study measured the effects of the interventions over three months, a longer follow-up period would provide more insight into the sustainability of benefits over time. Third, the study relied on self-reported measures that may be subject to response bias. Incorporating objective physiological or behavioral measures could provide a more comprehensive assessment of the intervention outcomes.

Several demographic characteristics differed significantly between the groups at baseline. Although linear mixed models accounted for repeated measures, multivariable adjustment for demographic covariates and propensity score methods were not performed because of the exploratory nature of the study and the relatively modest sample size. Therefore, residual confounding cannot be ruled out, and causal interpretations should be made cautiously. Additionally, differences in academic pressure across grade levels may have influenced baseline psychological distress levels, potentially affecting the group comparisons.

## Future directions

Future research should explore several key areas to enhance the effectiveness and sustainability of mindfulness- and yoga-based interventions for adolescents. First, longitudinal studies with extended follow-up periods are needed to assess the long-term retention of benefits and to identify strategies to sustain positive outcomes over time. Second, incorporating objective measures, such as physiological indicators (e.g., heart rate variability and cortisol levels) and behavioral assessments, could provide a more comprehensive understanding of the impact of these interventions beyond self-reported outcomes.

Future research should evaluate the implementation and effectiveness of the MYM intervention across multiple schools and diverse cultural and socioeconomic contexts. Large-scale cluster randomized trials with extended follow-up periods are required to establish the long-term effectiveness, cost-effectiveness, and scalability of this approach. Additionally, developing standardized teacher-training modules and digital support platforms may facilitate the wider dissemination and integration of these programs into school mental health programs.

## Conclusion

This quasi-experimental study provides preliminary evidence supporting the feasibility, acceptability, and potential effectiveness of both Mindful Yoga and Meditation (MYM) and the Common Yoga Protocol (CYP) in promoting adolescent mental health. While both interventions demonstrated significant benefits, the MYM group exhibited slightly greater improvements and sustained effects over time, likely due to the structured integration of mindfulness principles and real-life applications. The findings highlight the potential of integrating mindfulness-based yoga into school curricula as a feasible and cost-effective approach to supporting adolescent mental health. Given the observed decline in the intervention benefits over time, strategies to encourage long-term practice, such as incorporating brief mindfulness sessions into daily school routines, should be considered. Larger randomized studies with multivariate adjustments and longer follow-ups are needed to confirm these observations and establish the effectiveness of school-based mindfulness and yoga interventions.

## Supporting information

S1 File Bonferroni-Adjusted Pairwise Comparisons. (DOCX)
