# Supplementary material for: The effectiveness of a Mindful Yoga and Common Yoga intervention on emotion regulation and well-being of adolescents; A nonrandomized quasi-experimental study
Source: PLoS One. 2026 Aug 26;21(8):e0355375. doi: 10.1371/journal.pone.0355375 (PMC13514015; doi:10.1371/journal.pone.0355375)
Supplement: Supplementary file 1 [file pone.0355375.s001.docx]

**Bonferroni-Adjusted Pairwise Comparisons**

**Bonferroni-Adjusted Pairwise Comparisons for the Group × Time Interaction (K10 Scores)**

| Comparison | | | | |  |  |  |
| --- | --- | --- | --- | --- | --- | --- | --- |
| Group | **Time** |  | **Group** | **Time** | **Difference** | **SE** | **P _Bonferroni_** |
| C | 1 | - | C | 2 | 0.9574 | 0.572 | 1.000 |
| C | 1 | - | C | 3 | 0.0851 | 0.572 | 1.000 |
| C | 1 | - | CYP | 1 | 3.2610 | 0.958 | 0.028 |
| C | 1 | - | CYP | 2 | 8.2832 | 0.958 | <.001 |
| C | 1 | - | CYP | 3 | 5.7499 | 0.958 | <.001 |
| C | 1 | - | MYM | 1 | 4.4537 | 0.953 | <.001 |
| C | 1 | - | MYM | 2 | 8.6711 | 0.953 | <.001 |
| C | 1 | - | MYM | 3 | 8.5190 | 0.953 | <.001 |
| C | 2 | - | C | 3 | -0.8723 | 0.572 | 1.000 |
| C | 2 | - | CYP | 2 | 7.3258 | 0.958 | <.001 |
| C | 2 | - | CYP | 3 | 4.7924 | 0.958 | <.001 |
| C | 2 | - | MYM | 2 | 7.7137 | 0.953 | <.001 |
| C | 2 | - | MYM | 3 | 7.5615 | 0.953 | <.001 |
| C | 3 | - | CYP | 3 | 5.6648 | 0.958 | <.001 |
| C | 3 | - | MYM | 3 | 8.4339 | 0.953 | <.001 |
| CYP | 1 | - | C | 2 | -2.3035 | 0.958 | 0.612 |
| CYP | 1 | - | C | 3 | -3.1759 | 0.958 | 0.038 |
| CYP | 1 | - | CYP | 2 | 5.0222 | 0.585 | <.001 |
| CYP | 1 | - | CYP | 3 | 2.4889 | 0.585 | 0.001 |
| CYP | 1 | - | MYM | 1 | 1.1928 | 0.963 | 1.000 |
| CYP | 1 | - | MYM | 2 | 5.4101 | 0.963 | <.001 |
| CYP | 1 | - | MYM | 3 | 5.2580 | 0.963 | <.001 |
| CYP | 2 | - | C | 3 | -8.1981 | 0.958 | <.001 |
| CYP | 2 | - | CYP | 3 | -2.5333 | 0.585 | <.001 |
| CYP | 2 | - | MYM | 2 | 0.3879 | 0.963 | 1.000 |
| CYP | 2 | - | MYM | 3 | 0.2357 | 0.963 | 1.000 |
| CYP | 3 | - | MYM | 3 | 2.7691 | 0.963 | 0.159 |
| MYM | 1 | - | C | 2 | -3.4963 | 0.953 | 0.011 |
| MYM | 1 | - | C | 3 | -4.3686 | 0.953 | <.001 |
| MYM | 1 | - | CYP | 2 | 3.8295 | 0.963 | 0.003 |
| MYM | 1 | - | CYP | 3 | 1.2961 | 0.963 | 1.000 |
| MYM | 1 | - | MYM | 2 | 4.2174 | 0.578 | <.001 |
| MYM | 1 | - | MYM | 3 | 4.0652 | 0.578 | <.001 |
| MYM | 2 | - | C | 3 | -8.5860 | 0.953 | <.001 |
| MYM | 2 | - | CYP | 3 | -2.9213 | 0.963 | 0.097 |
| MYM | 2 | - | MYM | 3 | -0.1522 | 0.578 | 1.000 |

**Bonferroni-Adjusted Pairwise Comparisons for the Group × Time Interaction (CAMM Score)**

| Comparison | | | | |  |  |  |
| --- | --- | --- | --- | --- | --- | --- | --- |
| Group | **Time** |  | **Group** | **Time** | **Difference** | **SE** | **P _Bonferroni_** |
| C | 1 | - | C | 2 | -0.1277 | 0.547 | 1.000 |
| C | 1 | - | C | 3 | -0.4681 | 0.547 | 1.000 |
| C | 1 | - | CYP | 1 | 2.6052 | 0.985 | 0.316 |
| C | 1 | - | CYP | 2 | -0.0615 | 0.985 | 1.000 |
| C | 1 | - | CYP | 3 | 1.1830 | 0.985 | 1.000 |
| C | 1 | - | MYM | 1 | -1.4866 | 0.979 | 1.000 |
| C | 1 | - | MYM | 2 | -4.3562 | 0.979 | <.001 |
| C | 1 | - | MYM | 3 | -3.9431 | 0.979 | 0.003 |
| C | 2 | - | C | 3 | -0.3404 | 0.547 | 1.000 |
| C | 2 | - | CYP | 2 | 0.0662 | 0.985 | 1.000 |
| C | 2 | - | CYP | 3 | 1.3106 | 0.985 | 1.000 |
| C | 2 | - | MYM | 2 | -4.2285 | 0.979 | <.001 |
| C | 2 | - | MYM | 3 | -3.8154 | 0.979 | 0.005 |
| C | 3 | - | CYP | 3 | 1.6511 | 0.985 | 1.000 |
| C | 3 | - | MYM | 3 | -3.4750 | 0.979 | 0.017 |
| CYP | 1 | - | C | 2 | -2.7329 | 0.985 | 0.216 |
| CYP | 1 | - | C | 3 | -3.0733 | 0.985 | 0.074 |
| CYP | 1 | - | CYP | 2 | -2.6667 | 0.559 | <.001 |
| CYP | 1 | - | CYP | 3 | -1.4222 | 0.559 | 0.417 |
| CYP | 1 | - | MYM | 1 | -4.0918 | 0.990 | 0.002 |
| CYP | 1 | - | MYM | 2 | -6.9614 | 0.990 | <.001 |
| CYP | 1 | - | MYM | 3 | -6.5483 | 0.990 | <.001 |
| CYP | 2 | - | C | 3 | -0.4066 | 0.985 | 1.000 |
| CYP | 2 | - | CYP | 3 | 1.2444 | 0.559 | 0.970 |
| CYP | 2 | - | MYM | 2 | -4.2947 | 0.990 | <.001 |
| CYP | 2 | - | MYM | 3 | -3.8816 | 0.990 | 0.004 |
| CYP | 3 | - | MYM | 3 | -5.1261 | 0.990 | <.001 |
| MYM | 1 | - | C | 2 | 1.3589 | 0.979 | 1.000 |
| MYM | 1 | - | C | 3 | 1.0185 | 0.979 | 1.000 |
| MYM | 1 | - | CYP | 2 | 1.4251 | 0.990 | 1.000 |
| MYM | 1 | - | CYP | 3 | 2.6696 | 0.990 | 0.273 |
| MYM | 1 | - | MYM | 2 | -2.8696 | 0.553 | <.001 |
| MYM | 1 | - | MYM | 3 | -2.4565 | 0.553 | <.001 |
| MYM | 2 | - | C | 3 | 3.8881 | 0.979 | 0.004 |
| MYM | 2 | - | CYP | 3 | 5.5391 | 0.990 | <.001 |
| MYM | 2 | - | MYM | 3 | 0.4130 | 0.553 | 1.000 |

**Bonferroni-Adjusted Pairwise Comparisons for the Group × Time Interaction (ERQCR score)**

| Comparison | | | | |  |  |  |
| --- | --- | --- | --- | --- | --- | --- | --- |
| Group | **Time** |  | **Group** | **Time** | **Difference** | **SE** | **P _Bonferroni_** |
| C | 1 | - | C | 2 | 0.4681 | 0.314 | 1.000 |
| C | 1 | - | C | 3 | 0.8298 | 0.314 | 0.314 |
| C | 1 | - | CYP | 1 | 2.8203 | 0.615 | <.001 |
| C | 1 | - | CYP | 2 | 1.8426 | 0.615 | 0.111 |
| C | 1 | - | CYP | 3 | 2.5759 | 0.615 | 0.002 |
| C | 1 | - | MYM | 1 | 0.5208 | 0.612 | 1.000 |
| C | 1 | - | MYM | 2 | -1.6314 | 0.612 | 0.298 |
| C | 1 | - | MYM | 3 | -1.3270 | 0.612 | 1.000 |
| C | 2 | - | C | 3 | 0.3617 | 0.314 | 1.000 |
| C | 2 | - | CYP | 2 | 1.3745 | 0.615 | 0.956 |
| C | 2 | - | CYP | 3 | 2.1078 | 0.615 | 0.027 |
| C | 2 | - | MYM | 2 | -2.0994 | 0.612 | 0.026 |
| C | 2 | - | MYM | 3 | -1.7951 | 0.612 | 0.134 |
| C | 3 | - | CYP | 3 | 1.7461 | 0.615 | 0.180 |
| C | 3 | - | MYM | 3 | -2.1568 | 0.612 | 0.019 |
| CYP | 1 | - | C | 2 | -2.3522 | 0.615 | 0.006 |
| CYP | 1 | - | C | 3 | -1.9905 | 0.615 | 0.051 |
| CYP | 1 | - | CYP | 2 | -0.9778 | 0.321 | 0.091 |
| CYP | 1 | - | CYP | 3 | -0.2444 | 0.321 | 1.000 |
| CYP | 1 | - | MYM | 1 | -2.2995 | 0.618 | 0.009 |
| CYP | 1 | - | MYM | 2 | -4.4517 | 0.618 | <.001 |
| CYP | 1 | - | MYM | 3 | -4.1473 | 0.618 | <.001 |
| CYP | 2 | - | C | 3 | -1.0128 | 0.615 | 1.000 |
| CYP | 2 | - | CYP | 3 | 0.7333 | 0.321 | 0.831 |
| CYP | 2 | - | MYM | 2 | -3.4739 | 0.618 | <.001 |
| CYP | 2 | - | MYM | 3 | -3.1696 | 0.618 | <.001 |
| CYP | 3 | - | MYM | 3 | -3.9029 | 0.618 | <.001 |
| MYM | 1 | - | C | 2 | -0.0527 | 0.612 | 1.000 |
| MYM | 1 | - | C | 3 | 0.3090 | 0.612 | 1.000 |
| MYM | 1 | - | CYP | 2 | 1.3217 | 0.618 | 1.000 |
| MYM | 1 | - | CYP | 3 | 2.0551 | 0.618 | 0.038 |
| MYM | 1 | - | MYM | 2 | -2.1522 | 0.317 | <.001 |
| MYM | 1 | - | MYM | 3 | -1.8478 | 0.317 | <.001 |
| MYM | 2 | - | C | 3 | 2.4611 | 0.612 | 0.003 |
| MYM | 2 | - | CYP | 3 | 4.2072 | 0.618 | <.001 |
| MYM | 2 | - | MYM | 3 | 0.3043 | 0.317 | 1.000 |

**Bonferroni-Adjusted Pairwise Comparisons for the Group × Time Interaction (ERQES score)**

| Comparison | | | | |  |  |  |
| --- | --- | --- | --- | --- | --- | --- | --- |
| Group | **Time** |  | **Group** | **Time** | **Difference** | **SE** | **P _Bonferroni_** |
| C | 1 | - | C | 2 | 0.2128 | 0.297 | 1.000 |
| C | 1 | - | C | 3 | -0.3617 | 0.297 | 1.000 |
| C | 1 | - | CYP | 1 | 0.9820 | 0.498 | 1.000 |
| C | 1 | - | CYP | 2 | 1.8931 | 0.498 | 0.007 |
| C | 1 | - | CYP | 3 | 1.5820 | 0.498 | 0.061 |
| C | 1 | - | MYM | 1 | 0.4043 | 0.495 | 1.000 |
| C | 1 | - | MYM | 2 | 2.3173 | 0.495 | <.001 |
| C | 1 | - | MYM | 3 | 2.2303 | 0.495 | <.001 |
| C | 2 | - | C | 3 | -0.5745 | 0.297 | 1.000 |
| C | 2 | - | CYP | 2 | 1.6804 | 0.498 | 0.031 |
| C | 2 | - | CYP | 3 | 1.3693 | 0.498 | 0.231 |
| C | 2 | - | MYM | 2 | 2.1045 | 0.495 | 0.001 |
| C | 2 | - | MYM | 3 | 2.0176 | 0.495 | 0.002 |
| C | 3 | - | CYP | 3 | 1.9437 | 0.498 | 0.004 |
| C | 3 | - | MYM | 3 | 2.5920 | 0.495 | <.001 |
| CYP | 1 | - | C | 2 | -0.7693 | 0.498 | 1.000 |
| CYP | 1 | - | C | 3 | -1.3437 | 0.498 | 0.269 |
| CYP | 1 | - | CYP | 2 | 0.9111 | 0.304 | 0.106 |
| CYP | 1 | - | CYP | 3 | 0.6000 | 0.304 | 1.000 |
| CYP | 1 | - | MYM | 1 | -0.5778 | 0.500 | 1.000 |
| CYP | 1 | - | MYM | 2 | 1.3353 | 0.500 | 0.294 |
| CYP | 1 | - | MYM | 3 | 1.2483 | 0.500 | 0.479 |
| CYP | 2 | - | C | 3 | -2.2548 | 0.498 | <.001 |
| CYP | 2 | - | CYP | 3 | -0.3111 | 0.304 | 1.000 |
| CYP | 2 | - | MYM | 2 | 0.4242 | 0.500 | 1.000 |
| CYP | 2 | - | MYM | 3 | 0.3372 | 0.500 | 1.000 |
| CYP | 3 | - | MYM | 3 | 0.6483 | 0.500 | 1.000 |
| MYM | 1 | - | C | 2 | -0.1915 | 0.495 | 1.000 |
| MYM | 1 | - | C | 3 | -0.7660 | 0.495 | 1.000 |
| MYM | 1 | - | CYP | 2 | 1.4889 | 0.500 | 0.117 |
| MYM | 1 | - | CYP | 3 | 1.1778 | 0.500 | 0.700 |
| MYM | 1 | - | MYM | 2 | 1.9130 | 0.300 | <.001 |
| MYM | 1 | - | MYM | 3 | 1.8261 | 0.300 | <.001 |
| MYM | 2 | - | C | 3 | -2.6790 | 0.495 | <.001 |
| MYM | 2 | - | CYP | 3 | -0.7353 | 0.500 | 1.000 |
| MYM | 2 | - | MYM | 3 | -0.0870 | 0.300 | 1.000 |

**Bonferroni-Adjusted Pairwise Comparisons for the Group × Time Interaction (WHO5 score)**

| Comparison | | | | |  |  |  |
| --- | --- | --- | --- | --- | --- | --- | --- |
| Group | **Time** |  | **Group** | **Time** | **Difference** | **SE** | **P _Bonferroni_** |
| C | 1 | - | C | 2 | -0.362 | 0.575 | 1.000 |
| C | 1 | - | C | 3 | -0.149 | 0.575 | 1.000 |
| C | 1 | - | CYP | 1 | 0.960 | 0.765 | 1.000 |
| C | 1 | - | CYP | 2 | -3.106 | 0.765 | 0.002 |
| C | 1 | - | CYP | 3 | -1.506 | 0.765 | 1.000 |
| C | 1 | - | MYM | 1 | -0.889 | 0.761 | 1.000 |
| C | 1 | - | MYM | 2 | -5.411 | 0.761 | <.001 |
| C | 1 | - | MYM | 3 | -4.563 | 0.761 | <.001 |
| C | 2 | - | C | 3 | 0.213 | 0.575 | 1.000 |
| C | 2 | - | CYP | 2 | -2.745 | 0.765 | 0.014 |
| C | 2 | - | CYP | 3 | -1.145 | 0.765 | 1.000 |
| C | 2 | - | MYM | 2 | -5.049 | 0.761 | <.001 |
| C | 2 | - | MYM | 3 | -4.201 | 0.761 | <.001 |
| C | 3 | - | CYP | 3 | -1.357 | 0.765 | 1.000 |
| C | 3 | - | MYM | 3 | -4.414 | 0.761 | <.001 |
| CYP | 1 | - | C | 2 | -1.322 | 0.765 | 1.000 |
| CYP | 1 | - | C | 3 | -1.109 | 0.765 | 1.000 |
| CYP | 1 | - | CYP | 2 | -4.067 | 0.588 | <.001 |
| CYP | 1 | - | CYP | 3 | -2.467 | 0.588 | 0.001 |
| CYP | 1 | - | MYM | 1 | -1.849 | 0.769 | 0.605 |
| CYP | 1 | - | MYM | 2 | -6.371 | 0.769 | <.001 |
| CYP | 1 | - | MYM | 3 | -5.523 | 0.769 | <.001 |
| CYP | 2 | - | C | 3 | 2.957 | 0.765 | 0.005 |
| CYP | 2 | - | CYP | 3 | 1.600 | 0.588 | 0.248 |
| CYP | 2 | - | MYM | 2 | -2.304 | 0.769 | 0.107 |
| CYP | 2 | - | MYM | 3 | -1.457 | 0.769 | 1.000 |
| CYP | 3 | - | MYM | 3 | -3.057 | 0.769 | 0.003 |
| MYM | 1 | - | C | 2 | 0.527 | 0.761 | 1.000 |
| MYM | 1 | - | C | 3 | 0.740 | 0.761 | 1.000 |
| MYM | 1 | - | CYP | 2 | -2.217 | 0.769 | 0.152 |
| MYM | 1 | - | CYP | 3 | -0.617 | 0.769 | 1.000 |
| MYM | 1 | - | MYM | 2 | -4.522 | 0.581 | <.001 |
| MYM | 1 | - | MYM | 3 | -3.674 | 0.581 | <.001 |
| MYM | 2 | - | C | 3 | 5.262 | 0.761 | <.001 |
| MYM | 2 | - | CYP | 3 | 3.904 | 0.769 | <.001 |
| MYM | 2 | - | MYM | 3 | 0.848 | 0.581 | 1.000 |
